# Use of the Cognitive Performance Test for Identifying Deficits in Hospitalized Older Adults

**DOI:** 10.1155/2012/638480

**Published:** 2012-05-14

**Authors:** Alison Douglas, Lori Letts, Kevin Eva, Julie Richardson

**Affiliations:** ^1^School of Rehabilitation Science, McMaster University, IAHS Building, Rm 402, 1400 Main Street West, Hamilton, ON, Canada L8S 1C7; ^2^Department of Rehabilitation Science, McMaster University, ON, Canada; ^3^Centre for Health Education Scholarship, Department of Medicine, University of British Columbia, BC, Canada V5Z 1M9

## Abstract

*Objectives*. The Cognitive Performance Test (CPT) is a functional assessment for persons with dementia. The study purpose was to evaluate the reliability, discriminant, and concurrent validity of the CPT. *Method*. The CPT was tested against other measures of cognition (Standardized Mini Mental Status Exam (SMMSE) and Assessment of Motor and Process Skills-Process scale (AMPS-Process)). Participants were persons 65 years and older admitted to a geriatric rehabilitation unit (*n* = 47). *Results*. The CPT correlated moderately with measures of cognition (SMMSE *r* = 0.47, AMPS-Process *r* = 0.53, *P* < 0.01), and ADL burden of care (FIM *r* = 0.32, *P* < 0.05). Scores were not affected by age, sex, years of education, motor skills, or comorbidities. The CPT differentiated between impaired and unimpaired individuals differently from other measures. *Conclusion*. While CPT appears related to other measures of cognition, test interpretation requires noting the variability between CPT scores and those measures.

## 1. Introduction

Cognitive impairment is one of the strongest predictors of institutionalization, increasing the risk by two and a half times [1]. There is likely to be an increase in institutionalization with the aging population and an associated individual and societal burden. Evidence-based care is a priority with this patient population for caregivers, families, individuals, and policy makers. 

 Cognitive impairment may be associated with various pathologies including Alzheimer's disease, vascular dementia, mixed dementias, or cognitive decline secondary to Parkinson's disease. The role of occupational therapy is to enable the performance of everyday activities and participation [2, 3]. For persons with cognitive deficits, there is impairment in the ability to perform some activities of daily living (ADL) (essential self-care tasks, for example, bathing, dressing, grooming) and instrumental activities of daily living (IADLs) (a secondary set of tasks required for independent living, for example, cooking, shopping) [4]. Occupational therapists who see people with cognitive deficits in hospitals are concerned with their ability to live independently and safely after discharge and to recommend the amount of assistance required [5, 6]. Occupational therapists need to use an evidence-based evaluation of the ability of persons with cognitive deficits to complete functional tasks.

The Cognitive Performance Test (CPT) was developed for the assessment of older adults with cognitive deficits [7]. It is designed to structure observations of the performance of persons on specific ADL and IADL tasks. The test is designed for administration by an occupational therapist (OT) and does not require specialized training. Guidance regarding test interpretation is intended to assist with treatment planning and to predict a person's need for assistance. The attributes of feasibility of cost, level of training, and administration time means it has potential for uptake amongst clinicians.

 Occupational therapy literature describes two primary approaches to assessing cognition: a “bottom-up” approach and “top-down” approach [6, 8, 9]. A “bottom-up” approach examines basic cognitive processing to infer a person's function. The CPT uses a “top-down” approach, relying on the observation of performance on everyday tasks to ascertain cognitive abilities. The CPT is not designed as a measure of skill on specified tasks (e.g., ability to manage medication) but as a measure of working memory in everyday function [7]. The CPT scores are assigned based on the amount of cueing required to complete the tasks. Scoring rules are designed with the purpose of reducing the influence of motor and sensory functions.

The evidence to support the reliability of the CPT is based on four studies. Test-retest reliability, over a four-week interval, was reported as Pearson's *r* = 0.89 (*n* = 36) [10]. Interrater reliability between two raters was *r* = 0.91 (*n* = 18) and kappa  = 0.98 between two raters of two clients on video [11]. Internal consistency reliability was high (*r* = 0.84 [10], *r* = 0.95 [11]). A review comparing measures for older adults rated this reliability evidence as “adequate” [12] based on there being only one to two well-designed studies for each type of reliability [13].

The CPT has been validated using concurrent validity with other measures of cognition and activities of daily living (ADL). Validity data for the CPT is based on three published studies [10, 11, 14], resulting in the evidence for validity being rated as “adequate” [13]. Concurrent validity was demonstrated with the MMSE (Pearson's *r* = 0.67, *n* = 36 [10]; *r* = 0.76–0.88, *n* = 60 [11]) and ADL function using the Routine Task Inventory (*r* = 0.91–0.96 [11]) or Self Care Performance Test (*r* = 0.78 [14]). It has been correlated with caregiver reported ADL function using the Routine Task Inventory-caregiver (*r* = 0.50–0.68 (*n* = 60) [11]). The CPT manual cites unpublished data to support a correlation with neuropsychological tests of planning, sequencing, and attention [15] (*n* = 100). Measures of episodic memory, language, and comorbidity were not significantly correlated with CPT scores [15]. Predictive validity was described in a study demonstrating that a cognitive level of 4.2 or lower was a significant predictor of institutionalization over 4 years of followup [16].

Given this evidence, the CPT shows promise and would benefit from further study to address gaps in validity data. The CPT is intended to be a measure of cognition; therefore, it should be expected to correlate with measures of cognition but not with demographic characteristics or other constructs such as motor skill and ADL. It is not known if CPT measures cognition independently of demographic characteristics of age, sex, and education level. Because the CPT focuses on fairly straightforward tasks common to everyday living, it is not anticipated that these variables would have an effect on CPT performance. If any of these affect the score, a person, who is designated impaired (or unimpaired) by the test, may actually be unimpaired (or impaired) compared to others, their age, sex, or education level, thereby reducing the validity of the test interpretation or requiring more normative data based on these sources of variation.

It is not known if CPT measures the construct of cognition separately from other constructs such as daily living skills, chronic medical conditions, and motor skills. The CPT author acknowledges that, although the test was designed to minimize the influence of motor skill, motor skill may influence scores and data are needed to examine this relationship [7]. Determination that the CPT score does not correlate with the measures of daily living skills, chronic medical conditions and motor skills would support its construct validity. As a measure of cognition, CPT scores are expected to vary independently of daily living skills, comorbidities, and motor skills.

Moreover, it has not been determined whether, compared to other measures of cognition, the CPT identifies persons who require assistance to live in the community. The test is designed to help identify persons who need assistance. Therefore, it is important to understand the accuracy with which it identifies persons who are or are not impaired compared to other measures of cognition (designation of impairment). The precision of designation of impairment is a measurement property of the tool related to how well it differentiates persons who are deemed impaired versus unimpaired according to the tool's definition of impaired [17]. This could also be called the decision rule [17]. A previous study examined the designation of impairment for a measure similar to the CPT, called the Large Allen Cognitive Levels [18]. The previous study found no relation between designation of impairment on the LACL and cognitive measures. Therefore, it is also important to determine whether the CPT designates impairment similarly to other measures of cognition. These assessment scores contribute to decisions around independent living, and, therefore, data are required to determine the influence of age, education, comorbidities, activities of daily living (ADL) status, and motor skills on CPT scores.

In sum, the objectives of this study were to

1. determine whether age, sex, and years of education correlate with CPT scores,
2. determine correlation of CPT scores with measures of
  - ADL burden of care (Functional Independence Measure (FIM)),
  - chronic medical illness burden (CIRS-G),
  - motor skills (motor scale of the Assessment of Motor and Process Skills (AMPS)),
3. determine concurrent validity of CPT scores with measures of
  - cognitive screening (Standardized Mini Mental Status Exam (SMMSE)),
  - process skills (process scale of AMPS),
4. determine the agreement of CPT with SMMSE and AMPS regarding decision rules defining the threshold for impairment.

It was hypothesized that the functional measure of cognition (CPT), would not be associated with age, sex, or years of education, nor would it strongly correlate with measures of ADL, chronic illness burden, or motor skills. It was hypothesized that the CPT would show significant correlations with cognitive screening (MMSE) and process skills (AMPS-Process) and would define the threshold of impairment similarly to the SMMSE and AMPS-Process scale.

## 2. Methods

The study used a cross-sectional design. The data for this study represented baseline measurements of a prospective study that examined the predictive validity of functional/cognitive measures to predict safety six months after discharge.

### 2.1. Participants

Participants were recruited from consecutive admissions to a geriatric rehabilitation unit at a large urban teaching hospital. Persons admitted to the geriatric rehabilitation unit were included in the study if they were (1) 65 years or older, (2) referred to occupational therapy, (3) English speaking, and (4) undergoing functional or cognitive assessment with the OT because of a suspicion of cognitive impairment (diagnosis of dementia not required). Persons were excluded if they were (1) exhibiting symptoms of delirium or (2) had a primary diagnosis of mental illness excluding dementia noted on the chart. Recruitment and consent processes followed ethics guidelines and were approved by the local health research ethics board.

### 2.2. Assessment Instruments

The Cognitive Performance Test (CPT) was part of usual care on the geriatric rehabilitation unit for this study. The test requires direct observation of an individual performing up to seven activities (Medbox, Dress, Shop, Toast, Phone, Wash, and Travel). Scoring is described for each subtest individually in the test manual. Overall, higher scores (5 or 6) are given when little cueing or demonstration is required and low scores (1) when cues, which are specifically described for each subtest, must be given for the patient to complete the task. Total administration time is approximately 1/2 hour, and test administration is made efficient by allowing elements of a subtask to be omitted if a person shows low functioning during the assessment. Directions for standardized cues are provided. Scores on each task are added and divided by the number of subtasks given to calculate an average task performance (total score). The total score is also converted into a “Cognitive Level” (lowest level represents the most impairment) that is based on theoretical understanding of function in stages of dementia [7].

 The Standardized Mini Mental Status Exam (SMMSE) is a screening test for dementia with the same test items as the Mini Mental Status Examination (MMSE) [19]. The SMMSE differs from the MMSE because it has “clear, unequivocal guidelines for administration” [20, page 12], which increase the intrarater reliability compared to the MMSE [21]. The SMMSE has been reported to have greater accuracy than both generic and specific capacity assessment tools to identify incapacity to complete an advanced directive according to a gold standard (Competency Clinic) [22]. The SMMSE is a widely used instrument and was part of usual care at the study site.

The purpose of the AMPS [23] is to observe the performance of daily living tasks of the client's choice (approximately 15-minute interview), for a wide range of ages and diagnostic groups. The client is observed performing two tasks (approximately 1 hour) and performance is scored on 15 motor skills (e.g., lifts, walks) and 20 process skills (e.g., initiates, sequences). The AMPS scores incorporate four facets of assessment: item complexity, rater severity, task difficulty, and participant ability. Ratings are entered into a computer program that uses Rasch analysis of the four facets to determine two independent overall scores: AMPS-motor and AMPS-Process scores. Each scale has a cutoff point above which indicates an independence level and below which indicates the need for assistance. Administration requires OT qualifications, a one-week training course and certification with ten patients to determine Rasch values for scoring. The data to support the psychometric properties of the AMPS are derived from numerous studies, which report excellent test-retest reliability, inter- and intrarater reliability, and validation with measures of ADL, IADL, and cognitive tests [12].

The Functional Independence Measure (FIM) involves observation by the clinician of the patient's level of independence in activities of daily living [24] and was a mandated measure at the study site. The purpose of the measure is to describe the degree of disability and burden of care [24]. The patient's usual level of independence is observed during the daily routine. Scores are assigned from one (total assist) to seven (complete independence) on 19 daily living items (e.g., toileting, dressing) and are accumulated to determine the overall score, which indicates the degree of help required. For the total FIM score, five studies report high interrater reliability (ICC = 0.8–0.99) and high internal consistency (alpha = 0.8–0.97) [25]. Validity has been described in 41 studies with older adults and have repeatedly shown correlation with assessments of daily living skills [25–27] including in persons over age 80 [28].

The Cumulative Illness Rating Scale-for Geriatrics (CIRS-G) is a measure of chronic medical illness burden for older adults [29]. Diagnoses in 14 categories (e.g., vascular, renal) are assigned a severity score from 1 (not severe) to 4 (severe). The index score is the total score divided by the number of comorbid conditions and is an indication of the severity of concurrent conditions. Data demonstrate high levels of reliability [29].

### 2.3. Procedures

The CPT was administered by the attending occupational therapist (OT) as part of routine care. The Standardized Mini Mental Status Exam (SMMSE) [20] and the Functional Independence Measure [24] were administered by clinical staff as part of routine care. The participants' age and CIRS-G scores were generated from chart review, and education level was asked of the participants after administration of study measures.

Therapists administered 6 of 7 tasks of the CPT. The therapists were not able to feasibly administer the “phone” task due to lack of a working phone for patient use, which is acceptable since the test manual states that one or two tasks may be eliminated from the test. The Assessment of Motor and Process Skills [23] was administered by an outside assessor who was blind to the score on the CPT. To ensure that completion of the AMPS followed the assessment protocol, the AMPS administrator was aware of the patient medical history and SMMSE score. The AMPS version 7 cut-point scores were used to indicate independence [30].

The CPT and SMMSE, as part of usual care, were administered within several days of admission to the rehabilitation unit. Due to the consent process and feasibility of administration by the outside assessor, time elapsed between the administration of the usual care measures (CPT, SMMSE and FIM) and the AMPS scales. The average number of days between administration of the CPT and AMPS was 4.7 days (SD = 2.7 days).

### 2.4. Analyses

Internal consistency reliability of the CPT was determined using Cronbach's alpha. Determination of the influence of age, sex, and education was conducted using linear regression with CPT score as the dependent variable and age, sex, and education as independent variables. Discriminant validity of the CPT compared with measures of ADL, chronic medical illness, motor skills, was analyzed using Pearson's *r* correlations. Concurrent validity with cognitive screening and process skills was also analyzed with Pearson's *r* correlations. For the final objective, ROC curves were calculated to examine the sensitivity and specificity of the CPT to predict “independence” according to the SMMSE and AMPS scale. The ROC curve was used to determine the cut-point, which maximized both sensitivity and specificity of the CPT. Using this cut-point, the strength of agreement between the CPT and both measures (SMMSE and AMPS-Process scale) for designation of “independence” was determined using the phi statistic.

## 3. Results

This study aimed to examine the validity of the CPT by describing the degree of confidence that clinicians can place on CPT scores to identify cognitive impairment in older adults and the interpretation of functional status in a hospital setting. The results contribute to evidence regarding the validity of the CPT as a measure of cognition. However, when considering designation of impairment, there were weak associations between the measures.  Table 1 describes the sample demographics. The sample (*n* = 47) was composed of a majority of women (55.3%). Participants had a mean age of 83.5 years with 14 participants ≥85 years. A majority of participants (87.2%) had either grade school or high school education with a minority (12.8%) having postsecondary education.

The internal consistency of the CPT was acceptable (Cronbach's alpha = 0.71). The subtest-total correlation was acceptable at *r* > 0.2 for each subtest total score compared to the overall test score. Subtest-total statistics demonstrated that no subtest acted uniquely relative to any other. The ability of any single subtest to predict the scores on other subtests was low (ICC = 0.3).

CPT scores were not significantly influenced by age, sex, or years of education. This was shown by linear regression with CPT as the dependent variable and age, sex, and years of education as independent variables. This model had an overall *F*-test that was not significant (*F*(df = 2) = 0.47, *P* = 0.63). 

Correlations between scores on the CPT and validation measures revealed the anticipated pattern (see Table 2). When testing discriminant validity, a nonsignificant correlation was found with measures of motor skills (AMPS-Motor: *r* = 0.15, *P* = 0.32) and chronic medical illness (CIRS-G: *r* = −0.26, *P* = 0.08). There was a weak correlation with the measure of ADL burden (FIM: *r* = 0.32, *P* < 0.05). When testing concurrent validity, significant correlations were found with cognitive screen (SMMSE: *r* = 0.47, *P* < 0.01) and process skills (AMPS-Process: *r* = 0.53, *P* < 0.01). 

**Figure 1:**
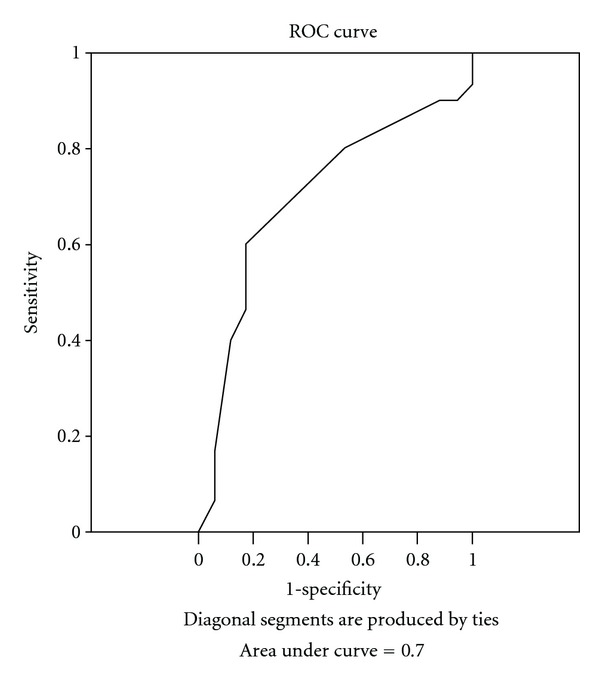
ROC curve for CPT with SMMSE as criterion.

**Figure 2:**
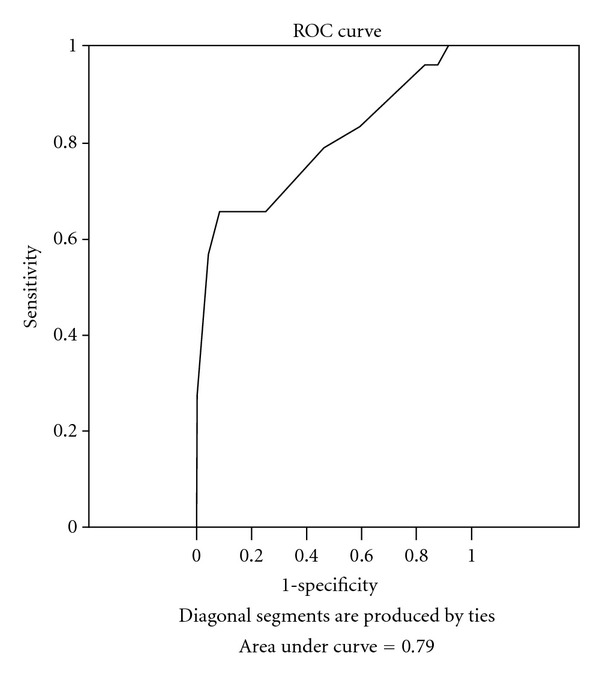
ROC curve for CPT with AMPS-Process as criterion.

The ability of the CPT to predict a designation of functional impairment on the SMMSE and AMPS was examined using ROC curves. The areas under the ROC curve were similar when comparing the CPT to the SMMSE (Area under curve = 0.70) and AMPS-Process scale (Area under curve = 0.79) (Figures 1 and 2). The sensitivity and specificity of the CPT for predicting impairment according to the SMMSE was optimized at a CPT cut-point of 4.5. At this point, the sensitivity of the CPT was 0.53 and the specificity was 0.82. The cut point that optimized sensitivity and specificity related to the AMPS was a CPT score of 4.6. At this point, the sensitivity was 0.75 and specificity was 0.65. 

Using the CPT cut score generated from the ROC curve, the overall agreement between the CPT and two criterion measures (SMMSE and AMPS-Process) was examined using the phi statistic. Results are shown in Tables 3 and 4. The strength of the association (phi statistic) was statistically significant when comparing the CPT to the SMMSE and AMPS-Process scale.

## 4. Discussion

### 4.1. Internal Consistency

The internal consistency of the CPT was acceptable, indicating that subtest scores were reasonably well correlated with one another. The single-measure intraclass correlation coefficient indicated that one could not reliably predict another subtest score from any single subtest and that multiple subtests need to be administered in order to obtain acceptable internal consistency. Therapists administering the CPT need to consider that there is a cost associated with reducing the number of subtests for clinical feasibility, in that the reliability of the overall score is reduced. Whenever possible, to maximize reliability, therapists should administer all of the CPT subtests. 

### 4.2. Lack of Effect of Age, Sex, and Number of Years of Education

In this study, age, sex, and number of years of education did not significantly relate to scores on the CPT. The scores did not reflect educational attainment nor were they affected by declines in age-related factors such as sensory decline or mental speed of processing. Additionally, it is valuable for a measure of cognition in daily living to be minimally influenced by sex and educational attainment. Many bottom-up cognitive tests (e.g., MMSE) are susceptible to the effects of education, and thus it might be argued that this finding indicates that the CPT was insensitive to cognitive differences related to age and education. Given that the test is a functional test of everyday actions performed by many people at all socioeconomic levels, the lack of effect of education may be indicative of an insensitive test or conversely of a test design that minimizes the effects of education. Thus, the score will more accurately reflect a person's ability to perform tasks on a daily basis (regardless of age, gender, or education) than the ability to successfully negotiate an assessment or testing environment. If a measure is influenced by education, persons with lower levels of education may be unfairly disadvantaged. These data indicate that the CPT was performing in this sample independently of age, sex, and educational attainment.

### 4.3. Lack of Correlation with Motor Skills and Comorbidities

The CPT was designed as a measure of cognition, and the lack of correlation with a measure of motor skills or comorbidities provides evidence of discriminant validity for the CPT, indicating that it is not principally measuring motor skills or physical factors related to diagnosis. The subtests of the CPT include daily living tasks such as washing hands and making toast. These require physical motor skills such as postural control, grasp, and hand coordination, but any influence that physical motor skills may have had on the scores of the CPT were not observable in this sample. The instructions for CPT administration specify ways to minimize the effects of motor ability on the overall score of CPT by, for example, moving the toaster closer to the person if it is too difficult to reach. The author of the CPT intended to reduce the effect of motor skills on scoring, and the data from this study indicate that, indeed, any effect of motor skills on the scoring is minimal. A number of comorbidities may be expected to influence cognition, such as vascular disease affecting brain vessels. The data from this sample did not show an influence of comorbidities on the score. This result requires further study with collection of data on both number and types of comorbidities to determine whether particular pathologies influence the score. 

The correlation with ADL burden of care (FIM) was weaker than the correlation with cognitive screening or process measures but stronger than with measures of motor skills and co-morbidities. This can be explained because FIM scores are influenced by both cognitive and motor skills. The FIM describes independence in tasks such as dressing and washing, such that those with a lower level of cognition require assistance and score lower on the FIM. Compared with measures of motor skill alone, FIM scores should therefore, be more highly correlated with CPT. However, compared with measures of cognition alone, FIM scores are influenced by motor skill, thus weakening the correlation with CPT.

### 4.4. Correlation with Cognitive Screen and Process Skills

The validity of the CPT was supported by statistically significant correlation with measures of cognitive screening (SMMSE) and process skills (AMPS Process scale), which may indicate that each is measuring a similar construct. The finding of statistically significant correlation between the CPT and SMMSE is similar to previous findings of correlation with the MMSE [10, 11]. However, the findings in this study show weaker correlation than the previous studies. Because the reliability of both measures is reported to be high (CPT *r* > 0.8 [10]; SMMSE *r* > 0.8 [21]), the correlation when corrected for reliability remains moderate. The SMMSE is designed to measure cognitive components including memory, attention, and abstraction, whereas the CPT examines working memory using daily living tasks. Furthermore, the SMMSE is designed as a screening tool, and the CPT is designed to give information about the level of cueing needed to do a task with the aim of treatment planning. The moderate correlation between scores may be explained by differences between the tools in overall design and purpose heterogeneity of the sample in terms of etiology of cognitive deficits (e.g., vascular dementia, Alzheimer's disease) or measurement error. 

The statistically significant correlation with the AMPS-Process scale provides evidence that the CPT is a measure of cognition. The AMPS-Process scale is correlated with measures of cognition including the LOTCA, Cognistat, and Rey Complex Figure-copy [31]. Correlation with the AMPS-Process scale adds to previous evidence suggesting a correlation between CPT and neuropsychological measures of planning, sequencing, and attention [15]. The finding of a moderate correlation suggests that the CPT and AMPS-Process may be unique in their measurement of cognition. A primary difference may lie in the construction and scoring of the measures. The CPT scoring is based on the amount of cueing required to complete tasks [10]. Scoring for the AMPS is based on observable actions, such as placement of items in the work space and task sequence. Another difference that may affect measurement is that the same CPT tasks are administered consistently, whereas the AMPS tasks are chosen by the patient. Although both use everyday tasks, the act of choosing a task in the AMPS may increase attention to the task, care in task completion, and perseverance in the face of difficulty.

### 4.5. Designation of Impairment Compared to Other Measures

The ability of the CPT to predict whether a person would score “impaired” on the AMPS-Process and SMMSE was examined using the cutoff points for the SMMSE and AMPS-Process scale. The area under the ROC curves (Figures 1 and 2) was similar when comparing the CPT to AMPS-Process scale and the CPT to SMMSE. This indicates that agreement between CPT scores and the designation of “independent” on the AMPS-Process scale was similar to the agreement with the SMMSE. 

The CPT was similar in designation of impairment compared to both criterion measures (SMMSE and AMPS-Process) according to the statistically significant phi coefficients. This finding differs from data comparing a measure similar to the CPT with the AMPS-Process scale [18]. Scoring of the CPT is based on the cognitive levels described in the test manual for the Large Allen Cognitive Levels Test (LACL) [18]. Therefore, the difference in findings is important to note. In the previous study, no relation was found between the LACL and AMPS-Process cutoff scores. The authors concluded that the LACL and AMPS differed in constructs being assessed. The finding of a relationship between CPT and AMPS in this study may indicate that the constructs assessed do not differ as much as previously concluded. The finding of a relationship between CPT and AMPS in this study may be due to use of a cut-point using ROC curves, whereas the previous study used three separate groups for CPT and two groups for AMPS in the analysis. Both studies reported similar sample demographics and range in scores. This current study was able to demonstrate an association with AMPS and therefore provide construct validation evidence for the CPT. 

It is important to note, however, that although the association was statistically significant in distinguishing impaired from unimpaired persons, the strength of the association was weak (phi coefficient less than 0.5). The degree of agreement between the CPT and SMMSE was higher for those who were not impaired. That is, persons who scored poorly on the CPT were more likely to score impaired on the SMMSE or AMPS-Process Scale. However, the sensitivity and specificity for detection of impairment was not consistent in all cases in that some persons who scored impaired on one measure were unimpaired on the other and vice versa. The conservative conclusion is that there was error associated with measurement in both instruments that resulted in differing designations of impairment. 

It must be noted that “impairment” on the CPT relates to functional impairment in daily living skills. The use of an actual diagnosis of dementia versus nondementia based on DSM-diagnostic criteria would be a useful gold standard to examine each measure for its accuracy as a diagnostic tool for dementia. As discussed later, a measure of safety and independence would be a valuable gold standard against which to examine the measures. 

When considering the use of the CPT in clinical practice, it is important to examine the number of persons who are designated as impaired by the measure. This informs the user about whether one measure was consistently more stringent at designating impairment than another. In this study, the CPT was not consistent with the SMMSE or AMPS-Process scale in designating impairment. This means that whether a person is identified as having impairment may depend on the measure chosen by the therapist, but also on error associated with measurement. The pattern of designation of impairment was not consistent in all cases. Clinical interpretation of the results must take into account the degree of uncertainty in identifying impairment. This study provides data that support the construct validation of the CPT and test scores must be interpreted alongside other clinical observation of cognition and function when working with people with dementia.

### 4.6. Limitations and Directions for Future Research

The range of CPT scores in the sample was restricted from 3.8 to 5.5. Restriction in range with a high number of persons at the middle range of the scores places a limit on finding a strong correlation [17]. The sample was drawn from an inpatient rehabilitation unit and although potentially representative of geriatric inpatients, was not representative of community-dwelling older adults. Further restriction in range was avoided by not requiring the sample to have a diagnosis of dementia, merely a suspicion of cognitive deficit, and persons who were not suspected to have cognitive deficits were not on the caseload of the clinicians administering the test measures. The sample also may have included different dementia etiologies (e.g., vascular dementia, Alzheimer's disease). Correlations may have been stronger if there had been a greater range in CPT scores. 

A higher alpha coefficient for internal consistency may have been obtained if all subtests had been administered; however, this was not feasible in the clinical setting and the alpha level obtained was acceptable. The CPT test was administered in the course of usual care thus therapist administrator and time may be confounding the correlation between measures. Two therapists administered the CPT, and although they both had training given by the CPT author, interrater reliability was not established and may have reduced the strength of the correlations found. Also, clinical feasibility meant that there was a time lag of days (average 4.7 days) between measures. This time lag was similar to a previous study [31], the participants were medically stable, and they did not display symptoms of delirium. It is likely that the participants would not have tolerated administration of all measures on a single day, and fatigue could negatively influence test performance. The correlations were significant as hypothesized, but they may have been stronger if the measures had been administered on the same day or closer together in time. 

Further research is required to assess the predictive validity of the CPT to determine safety at home upon discharge from rehabilitation. Occupational therapists need “top-down” performance-based measures for predicting function and making recommendations for discharge from rehabilitation. There is a call for cognitive tests that are ecologically relevant, and based on real-world performance, as they will provide an empirical foundation for decisions on safety [32]. The CPT, as a performance-based test, is well situated to meet this need. Moreover, because the CPT examines both cognitive capacities and daily function, it can be used for both identifying deficits and predicting function. Therefore, it is attractive as an “efficient” measure [33]. This study demonstrates that the CPT is a valid measure of cognition that was not influenced by age, sex, education, or motor function. The interpretation of the score must be accompanied by a statement regarding evidence for error in identifying impairment in a sample of older adults with cognitive impairment. Further development of occupational therapy assessments of function will promote ethical decision-making in the care of persons with dementia.

## Figures and Tables

**Table 1:** Description of sample.

	Valid (*N*)	Range	Mean	SD
Gender				
Male	21 (44.7%)			
Female	26 (55.3%)			
Age		66–97	83.5	7.7
Years of education		2–17	10.2	3.1
FIM score		76−119	103.09	12.25
CIRS-G		1.30–2.60	2.04	0.27
SMMSE score		10–30	23.79	4.05
AMPS-Motor		−0.25–2.24	0.95	0.54
AMPS-Process		−0.42−1.49	0.66	0.45
CPT score		3.8–5.5	4.53	0.38

**Table 2:** Pearson correlation coefficients of CPT with validation measures.

Measure	CPT	FIM	CIRS-G	AMPS-Motor	SMMSE	AMPS-Process
Cognitive Performance Test (CPT)	1.0	0.32*	−0.26	0.15	0.47**	0.53**
Functional Independence Measure (FIM)		1.0	−0.09	0.62**	0.19	0.33*
Cumulative Illness Rating Scale-Geriatric (CIRS-G)			1.0	−0.28	−0.38**	−0.22
AMPS-Motor				1.0	0.11	0.43**
Standardized Mini Mental Status Exam (SMMSE)					1.0	0.46**
AMPS- Process						1.0

*Significant at *P* < 0.05 level; **significant at *P* < 0.01 level.

**Table 3:** Designation of functional impairment: Phi test of CPT and SMMSE.

CPT total score	SMMSE designation		Total
	0–26 Impaired	>26 (Unimpaired)	
<4.5 (impaired)	16	3	19
4.5 or higher (unimpaired)	14	14	28

Total	30	17	47

Phi = 0.35; *P* = 0.02.

**Table 4:** Designation of functional impairment: Phi test of CPT and AMPS-process.

CPT total score	AMPS-Process designation		Total
	Dependent	Independent	
<4.6 (impaired)	18	8	26
4.6 or more (unimpaired)	6	15	21

Total	24	23	47

Phi = 0.40; *P* = 0.01.

## References

[1] Gaugler JE, Duval S, Anderson KA, Kane RL (2007). Predicting nursing home admission in the U.S: a meta-analysis. *BMC Geriatrics*.

[2] Crepeau EB, Cohn ES, Schell BB, Crepeau EB, Cohn ES, Boyt-Schell B (2003). Occupational therapy practice. *Occupational Therapy*.

[3] Townsend EA, Polatajko H (2007). *Enabling Occupation II: Advancing an Occupational Therapy Vision of Health, Well-Being And Justice Through Occupation*.

[4] Lawton MP (1971). The functional assessment of elderly people.. *Journal of the American Geriatrics Society*.

[5] Bonder BR, Wagner MB (2001). *Functional Performance in Older Adults*.

[6] Vining Radomski M, Trombly CA, Vining-Radomski M (2002). Assessing abilities and capacities: cognition. *Occupational Therapy for Physical Dysfunction*.

[7] Burns T (2006). *Cognitive Performance Test Manual*.

[8] Duchek JM, Abreu B, Christianson C, Baum C (1997). Meeting the challenges of cognitive disabilities. *Occupational Therapy: Enabling Function and Wellbeing*.

[9] Grieve J (2000). *Neuropsychology for Occupational Therapists*.

[10] Burns T, Allen CK, Earhart CA, Blue T (1992). The Cognitive Performance Test: an approach to cognitive level assessment in Alzheimer disease. *Occupational Therapy Treatment Goals for the Physically and Cognitively Disabled*.

[11] Bar-Yosef C, Weinblatt N, Katz N (1999). Reliability and validity of the Cognitive Performance Test (CPT) in an elderly population in Israel. *Physical and Occupational Therapy in Geriatrics*.

[12] Douglas A, Letts L, Liu L (2007). Review of cognitive assessments for older adults. *Physical and Occupational Therapy in Geriatrics*.

[13] Law M, Baum C, Dunn W (2005). *Measuring Occupational Performance: Supporting Best Practice in
Occupational Therapy*.

[14] Thralow JU, Reuter MS (1993). Activities of daily living and cognitive levels of function in dementia. *American Journal of Alzheimer’s Care and Related Disorders & Research*.

[15] Bares KK (1998). *Neuropsychological predictors of functional level in Alzheimer's disease*.

[16] Burns T, Mortimer JA, Merchak P (1994). Cognitive performance test: a new approach to functional assessment in Alzheimer’s disease. *Journal of Geriatric Psychiatry and Neurology*.

[17] Streiner DL, Norman GR (2003). *Health Measurement Scales: A Practical Guide to Their Development and Use*.

[18] Marom B, Jarus T, Josman N (2006). The relationship between the Assessment of Motor and Process Skills (AMPS) and the Large Allen Cognitive Level (LACL)test in clients with stroke. *Physical and Occupational Therapy in Geriatrics*.

[19] Folstein MF, Folstein SE, McHugh PR (1975). “Mini-Mental State”: a practical method for grading the cognitive state of patients for the clinician. *Journal of Psychiatric Research*.

[20] Molloy DW, Clarnette R (1999). *Standardized Mini Mental Status Exam (SMMSE): A User’s Guide*.

[21] Molloy DW, Alemayehu E, Roberts R (1991). Reliability of a standardized mini-mental state examination compared with the traditional mini-mental state examination. *American Journal of Psychiatry*.

[22] Molloy DW, Silberfeld M, Darzins P (1996). Measuring capacity to complete an advance directive. *Journal of the American Geriatrics Society*.

[23] Fisher AG (2006). *Assessment of Motor and Process Skills. Vol. 2: User Manual*.

[24] Uniform Data System for Medical Rehabilitation: Functional Independence Measure. http://www.udsmr.org/.

[25] Glenny C, Stolee P (2009). Comparing the functional independence measure and the inter RAI/MDS for use in the functional assessment of older adults: a review of the literature. *BMC Geriatrics*.

[26] Letts L, Bosch J, Law M, Baum C, Dunn W (2005). Measuring occupational performance in basic activities of daily living. *Measuring Occupational Performance: Supporting Best Practice in Occupational Therapy*.

[27] McDowell I, Newell C (1996). *Measuring Health: A Guide to Rating Scales and Questionnaires*.

[28] Pollak N, Rheault W, Stoecker JL (1996). Reliability and validity of the FIM for persons aged 80 years and above from a multilevel continuing care retirement community. *Archives of Physical Medicine and Rehabilitation*.

[29] Miller MD, Paradis CF, Houck PR (1992). Rating chronic medical illness burden in geropsychiatric practice and research: application of the Cumulative Illness Rating Scale. *Psychiatry Research*.

[30] Merritt BK (2010). Utilizing AMPS ability measures to predict level of community dependence. *Scandinavian Journal of Occupational Therapy*.

[31] Kizony R, Katz N (2002). Relationships between cognitive abilities and the process scale and skills of the assessment of motor and process skills (AMPS) in patients with stroke. *Occupational Therapy Journal of Research*.

[32] Burgess PW, Alderman N, Forbes C (2006). The case for the development and use of "ecologically valid" measures of executive function in experimental and clinical neuropsychology. *Journal of the International Neuropsychological Society*.

[33] Milberg W (1996). Issues in the assessment of cognitive function in dementia. *Brain and Cognition*.

